# A QUALITY INITIATIVE TO INCREASE HOSPICE REFERRALS FOR STARS PATIENTS DURING THE SECOND COVID SURGE

**DOI:** 10.1093/geroni/igac059.2749

**Published:** 2022-12-20

**Authors:** Perihan El Shanawany, Chris Nouryan, Chris Choi, Ann Flynn, Edith Burns

**Affiliations:** Northwell Health, Plainview, New York, United States; Zucker School of Medicine at Hofstra Northwell, Uniondale, New York, United States; Northwell Health, Uniondale, New York, United States; Northwell Health, Uniondale, New York, United States; Zucker School of Medicine at Hofstra Northwell, Manhasset, New York, United States

## Abstract

**Background:**

CMS uses the Overall Hospital Quality Star Rating program to stratify hospitals based on specific quality criteria (e.g., 30-day readmissions of older adults with pneumonia). “STARS patients” experience more readmissions, longer LOS and often have more complex discharge plans. During the second surge of COVID we implemented a program to increase hospice referrals through early identification and implementation of goals of care (GOC) conversations.

**Methods:**

Electronic Medical Records reviewed for STARS patients from pre- (1/2019-7/2020) and post (3/2021–2/2022) program implementation. Location: 2 community-based hospitals. Data collected: demographics, hospital outcomes, discharge disposition. Data compared with Student’s t tests and Chi square.

**Results:**

459 patients, 177 pre-program and 282 post-program were included. Groups were similar in age (83.0 vs 83.6), LACE score (13.0 vs 12.8), principal diagnoses (PNA: 41.5% vs 46.0%, HF/COPD/AMI: 58.5% vs 54.0%), and mortality (3.5 vs. 4.0%). LOS increased 4.9 days vs. 6.1 days (p < 0.01), readmission rates unchanged: 12.6% vs 13.0% (p=0.90). GOC conversations increased, 48.6% to 75.0% (p < 0.01), DNR from 24% to 44% (p < 0.01), and comfort measures from 0.5% to 5% (p < 0.01). Hospice referrals increased from 0.5% to 11.2% (p < 0.01). Discussion: Early identification of STARS patients increased GOC conversations, DNR, comfort measures and hospice referral. Patients across time periods were similar in age, LACE and admitting diagnoses. LOS increased by a day, likely reflecting time needed to arrange discharge disposition. Increased hospice at end-of-life is associated with better quality care and patient/family satisfaction. This program may be adapted to larger, academic medical centers within the health system.

